# Anodal High-definition Transcranial Direct Current Stimulation Over the Left (but not Right) Parietal Cortex Facilitates Mental Arithmetic

**DOI:** 10.1007/s41465-024-00314-0

**Published:** 2024-12-04

**Authors:** Matthias Hartmann, Magali Dumureau

**Affiliations:** 1 Faculty of Psychology, UniDistance Suisse, Schinerstrasse 18, Brig, 3900 Switzerland; 2 Department of Psychology, University of Bern, Bern, Switzerland

**Keywords:** Mental arithmetic, tDCS, HD-tDCS, Posterior parietal cortex (PPC), Intraparietal sulcus (IPS), Numerical cognition

## Abstract

Non-invasive brain stimulation (NIBS) techniques such as transcranial direct current stimulation (tDCS) allow for investigating the functional involvement of specific brain areas in mental arithmetic. In this study, we employed for the first time high-definition (HD)-tDCS, which offers enhanced spatial precision, to explore the functional roles of the left and right intraparietal sulcus (IPS) in mental arithmetic. A total of 25 participants underwent anodal left IPS, anodal right IPS, and sham stimulation in separate sessions in counterbalanced order while solving single- and multi-step addition and subtraction problems. We found that stimulation of the left IPS, but not the right IPS or sham stimulation, improved arithmetic performance speed. These results provide further evidence for the functional involvement of the left IPS in a broad range of arithmetic tasks and highlight the potential of NIBS for cognitive enhancement.

**Supplementary Information:**

The online version contains supplementary material available at 10.1007/s41465-024-00314-0.

## Introduction

The ability to process numbers and perform mental arithmetic is a fundamental cognitive function, with mathematical skills strongly linked to academic achievement, career success, earning potential, and overall quality of life (Parsons & Bynner, 2005; Ritchie & Bates, 2013). As a result, neuroscientists have devoted substantial effort to investigating the neural and cognitive mechanisms underlying number processing and mental arithmetic, as well as exploring tools to support mathematical learning (Fischer et al., 2011; Park & Brannon, 2014; Snowball et al., 2013; Wilson et al., 2006). Neuroimaging studies have identified distinct neural networks associated with different types of number processing. For example, simple tasks like number comparisons primarily engage a parietal network, while more complex tasks such as mental arithmetic recruit a fronto-parietal network (Arsalidou & Taylor, 2011). The frontal regions within this network, including the middle and superior frontal gyri, are thought to reflect cognitive demands such as working memory and attentional processes required during calculation (Arsalidou & Taylor, 2011; Wilkey & Price, 2019). In contrast, the parietal component, particularly the intraparietal sulcus (IPS), serves as a key hub for magnitude representation and mathematical computations (Amalric & Dehaene, 2019; Castaldi et al., 2020; Dehaene, 2011; Eger et al., 2009; Karolis et al., 2019; Piazza et al., 2007; Salillas et al., 2023).

Neuroimaging techniques are limited in their ability to distinguish between epiphenomenal activity and critical, functionally relevant activity in brain areas during mental arithmetic. To overcome this limitation, non-invasive brain stimulation (NIBS) techniques provide a useful alternative. By directly modulating neural activity, NIBS allows researchers to gather more direct evidence regarding the involvement of specific brain regions in cognitive processes (Garcia-Sanz et al., 2022; Schroeder et al., 2017). A key source of evidence supporting the functional role of fronto-parietal areas in mental arithmetic comes from NIBS studies using transcranial magnetic stimulation (TMS) (Garcia-Sanz et al., 2022). TMS generates a magnetic field through a coil positioned on the skull, which induces an electric current that disrupts neuronal activity in the nearby brain regions. In TMS studies, inhibitory stimulation protocols are often used, leading to impairments in task performance and providing insight into the critical role of those regions in specific cognitive functions (Jahanshahi & Rothwell, 2000).

Despite numerous studies investigating the inhibitory effects of TMS on mental arithmetic, a clear understanding of the role of the IPS, particularly regarding lateralization (i.e., the contribution of the left versus right IPS), and its specific involvement in different aspects of arithmetic processing, remains elusive (Garcia-Sanz et al., 2022). Some studies have shown that stimulating the left IPS impairs arithmetic performance, while right IPS stimulation has no effect (Göbel et al., 2006). Conversely, other studies have reported no differences between left and right IPS stimulation (Montefinese et al., 2017, for multiplication problems; Salillas et al., 2012, for addition problems), or have indicated a stronger or selective impairment associated with right IPS stimulation (Montefinese et al., 2017, for addition problems; Salillas et al., 2012, for multiplication problems). However, there are also studies that found no TMS-related effects on arithmetic performance at all (Fresnoza et al., 2020). There is considerable variability in TMS methods across studies, including differences in pulse type and frequency (e.g., single vs. repetitive pulses), coil placement, stimulation intensity, and the timing of stimulation in relation to the arithmetic task. The influence of these factors is not yet fully understood but likely plays a role in the divergent findings reported in previous research (Garcia-Sanz et al., 2022; Hobot et al., 2021; Salillas et al., 2012).

The second line of evidence supporting the functional involvement of parietal brain areas in mental arithmetic comes from studies using transcranial electric stimulation (Fresnoza & Ischebeck, 2024; Lazzaro et al., 2022). Transcranial electric stimulation can be further divided into transcranial direct current stimulation (tDCS), transcranial alternating current stimulation (tACS), and transcranial random noise stimulation (tRNS). tDCS involves the application of a constant electrical current to the brain, typically between 0.5 to 2 mA, through anodal and cathodal electrodes placed on the scalp (Nitsche et al., 2008). Anodal tDCS is believed to increase cortical excitability by raising the resting membrane potential of neurons, bringing them closer to the threshold for activation without directly inducing action potentials (Bikson et al., 2016; Brezis et al., 2016). In contrast, cathodal tDCS is thought to reduce cortical excitability by lowering the resting membrane potential. While tDCS involves a constant electrical current, tACS uses alternating currents at specific frequencies to modulate neural oscillations in targeted brain areas, aiming to influence cognitive processes linked to these oscillatory patterns (Antal & Paulus, 2013). Finally, tRNS is a more recent technique that delivers random alternating currents to the cortex and may induce long-term effects (Terney et al., 2008). These transcranial electrical stimulation techniques are currently being explored as promising methods to enhance cognitive performance across various cognitive domains (Mancuso et al., 2016; Santarnecchi et al., 2015). In the context of mental arithmetic, most prior studies have used anodal tDCS to enhance concurrent arithmetic performance (Artemenko et al., 2015; Clemens et al., 2013; Hauser et al., 2013; Kasahara et al., 2013; Klein et al., 2013; Mosbacher et al., 2020; Rütsche et al., 2015). Some studies have also employed tACS or tRNS, primarily to support the learning of novel arithmetic facts and procedures over multiple sessions, with a focus on long-term improvement (Lazzaro et al., 2022, 2023; Mosbacher et al., 2021; Pasqualotto, 2016; Popescu et al., 2016; Snowball et al., 2013). Since the focus of the current study is not on arithmetic learning, we mainly concentrate on tDCS studies assessing short-term improvements in arithmetic performance through IPS stimulation.

In the first tDCS study demonstrating a beneficial effect of IPS stimulation on mental arithmetic, Hauser et al. (2013) reported improved performance for subtraction problems following anodal tDCS over the left IPS, whereas right IPS or bilateral stimulation did not yield similar benefits (see also Kasahara et al., 2013). This pattern was replicated in one follow-up study (Grabner et al., 2015) but not in others (Hauser et al., 2016; see also Mosbacher et al., 2020, 2021). Another study used bilateral tDCS with two electrodes of the same polarity to simultaneously stimulate the left and right IPS regions (Klein et al., 2013). While there were specific effects on number processing, no clear improvement in arithmetic performance was observed (see also Artemenko et al., 2015). The exact roles of the left and right IPS in mental arithmetic, as well as the potential of transcranial electric stimulation to enhance arithmetic performance, remains open questions.

One possible explanation for the inconsistent tDCS findings is the varying task demands across studies. Different arithmetic tasks, including addition, subtraction, or multiplication, have been used in previous research (Artemenko et al., 2015; Grabner et al., 2015; Hauser et al., 2013; Mosbacher et al., 2020). Each type of arithmetic operation is associated with distinct neural activity patterns (Andres et al., 2011; Arsalidou & Taylor, 2011; Castaldi et al., 2020; Menon, 2015). For example, addition and multiplication rely more on retrieval from long-term memory, potentially making them less dependent on the IPS compared to subtraction (Grabner et al., 2009; Yang et al., 2017). Additionally, within the same operation type, difficulty plays a significant role: More demanding problems, such as those requiring more computational steps or involve larger numbers, engage the fronto-parietal network to a greater degree due to increased manipulation of numerical quantity and working memory load (Metcalfe et al., 2013; Mosbacher et al., 2021; Tschentscher & Hauk, 2016; Wu et al., 2009; Yang et al., 2017). However, most prior tDCS studies have not considered more than one type of operation or have not accounted for differences in difficulty within the same operation type (for exceptions see Artemenko et al., 2015; Mosbacher et al., 2020).

There are also methodological differences in tDCS studies, such as the timing of stimulation (during or prior to the task), stimulation intensity, and electrode placement (Artemenko et al., 2015; Clemens et al., 2013; Hauser et al., 2013; Kasahara et al., 2013; Klein et al., 2013; Mosbacher et al., 2020; Rütsche et al., 2015). Most prior studies on mental arithmetic used traditional tDCS setups, where a relatively large anode (for enhancing effects) is placed over the IPS, and a cathode is positioned on the contralateral supraorbital region (Artemenko et al., 2015; Grabner et al., 2015; Hauser et al., 2013; Mosbacher et al., 2020). This configuration may introduce an unintended confound: the cathode, placed near the prefrontal cortex, could potentially inhibit prefrontal activity, which plays a crucial role in mental arithmetic. This inhibitory effect might counteract the benefits of anodal stimulation on the IPS. Similarly, tDCS over the IPS might affect neighboring regions, such as the angular gyrus, which is involved in arithmetic fact retrieval (Bloechle et al., 2016; Grabner et al., 2009; Hauser et al., 2016; Sokolowski et al., 2023), making it difficult to isolate IPS-specific effects. High-definition tDCS (HD-tDCS) offers a solution to these limitations by providing more focal stimulation through smaller electrodes (Datta et al., 2009; Kuo et al., 2013). HD-tDCS also splits the cathode into multiple smaller electrodes with sub-threshold activity, reducing the risk of unintended inhibitory effects. While HD-tDCS has recently been used to investigate the IPS in non-symbolic approximate arithmetic (Hartmann et al., 2020), it has yet to be applied to symbolic arithmetic.^1^

### The Present Study

Given the inconsistent findings regarding the specific contribution of the IPS to mental arithmetic and the effects of NIBS, further investigation is warranted. This study aims to clarify the functional involvement of the IPS in mental arithmetic and to further assess the potential of non-invasive electrical brain stimulation, while addressing limitations identified in previous research. First, this is, to our knowledge, the first study to employ HD-tDCS in this context, which reduces the likelihood of unintended co-activation in adjacent brain regions or inhibitory effects in distant areas, such as the prefrontal cortex. This allows for more precise targeting of the IPS (Datta et al., 2009; Kuo et al., 2013), potentially resulting in clearer and more robust cognitive enhancements. Second, to better understand how different arithmetic operations and problem difficulty may influence the potential enhancing effects of stimulation, we manipulated both the type of operation (addition vs. subtraction) and the problem complexity by varying the number of computational steps (one step vs. three steps).

Based on previous research, we hypothesized that anodal HD-tDCS, particularly over the left IPS, would enhance performance in mental arithmetic (Grabner et al., 2015; Hauser et al., 2013; Kasahara et al., 2013). Moreover, addition problems are typically solved faster than subtraction problems, as they are generally more familiar and rely more on automatic processes and memory retrieval (Campbell, 2008; Fayol & Thevenot, 2012; Thevenot et al., 2007). This increased automatization and reliance on memory retrieval may make addition less dependent on the magnitude-processing network in the IPS. Consequently, we further hypothesized that the effect of anodal HD-tDCS over the IPS would be stronger for subtraction compared to addition problems. Finally, as the number of computational steps increases, reliance on fact retrieval decreases while the need for calculation procedures grows (Hartmann, 2022; Tschentscher & Hauk, 2016). The prolonged cognitive demands of multi-step problems require continuous access to number meanings from the IPS and the maintenance of problem state representations, further amplifying reliance on the IPS (Anderson et al., 2007; Stocco et al., 2012). Therefore, we also hypothesized a stronger facilitatory effect of IPS stimulation for multi-step problems compared to single-step problems, as the stimulation effect may accumulate with increasing computational demands.

## Materials and Methods

### Ethics Approval

The study was approved by the Ethics Committee of the University of Bern and experiments were conducted in accordance with the ethical standards as laid down in the 1964 Declaration of Helsinki and its later amendments. Participants gave written informed consent prior to the study.

### Participants and Sample Size

Twenty-five healthy right-handed participants (17 female, 8 male) took part in this study (mean age: 23.9, ranging from 19–35). Participants either received course credit or monetary compensation (35 Swiss Francs) for their participation. All participants confirmed by self-report that they had no history of psychiatric or neurological disorders, did not take any drugs or abuse alcohol, and did not have developmental dyscalculia or any other impairment in number processing. These exclusion criteria were clearly defined in the experiment advertisement, and no individuals meeting these criteria volunteered to participate.

The number of participants was comparable to previous tDCS studies on mental arithmetic (Artemenko et al., 2025; Hauser et al., 2013; Klein et al., 2013; Mosbacher et al., 2020) and exceeded the minimum required sample size of *N* = 24, as suggested by G*Power (Faul et al., 2007) to detect a difference between left parietal anodal tDCS stimulation and sham stimulation on mental arithmetic speed, with 80% power and an alpha level of 0.05. This estimation was based on an effect size of d = 0.52, calculated from the *t*-value reported inHauser et al. (2013).^2^ We initially targeted a sample size of 24 to counterbalance stimulation (left, right, sham) across sessions (see below), but 25 participants signed up and we decided to include all participants in the study.

### Design, Randomization and Blinding

We used a 3 (HD-tDCS: left, right, sham) × 2 (operation: addition, subtraction) × 2 (step size: single, multi) single-blind sham-controlled within-participants design whereby each participant received left parietal, right parietal, and sham stimulation in separate sessions (one week between sessions). Each participant was randomly assigned to one of the six possible stimulation sequences generated from the three conditions (left, right, sham), with each sequence assigned to four participants (n = 24). One additional participant was then randomly assigned to one of the six sequences. Participants were informed that they would experience three different stimulation conditions, but they were not told the order of the conditions, nor that one of them was a sham. Additionally, they were unaware that performance improvement was specifically expected for the left HD-tDCS condition.

### HD-tDCS and Stimulation Procedure

Bieck et al (2018) recently applied parietal tRNS and did not observe any effects on arithmetic performance. They concluded that tRNS appears to have less influence on concurrent mental arithmetic compared to tDCS, particularly when applied over the IPS, and that the effects of tES may be more pronounced in training paradigms. Since the focus of this study is on the short-term effects of stimulation to better understand the role of the IPS in mental arithmetic, we opted to use tDCS rather than tRNS.

A 1-anode, 4-cathode electrode setting was used (4 × 1). HD-tDCS was administered using a battery-driven, constant-current generator with a HD-tDCS distributor (DC-Stimulator MC, neuroConn GmbH, Germany). All electrodes had a size of 1 cm in diameter and were attached to ordinary EEG-caps (Easycap GmbH, Germany). Participants’ hair under the electrode casings were moved aside to expose the scalp skin, and a conductive gel was injected into the electrode casings (Signa Gel, Parker Laboratories, NJ, USA).

The anodal stimulation was set to 2 mA, and consequently the cathodal stimulation was 0.5 mA per electrode. Previous studies also used 2 mA in order to modulate parietal activity during mental arithmetic (Clemens et al., 2013; Hartmann et al., 2020; Houser et al., 2015), and the tolerability of a 2 mA anodal stimulation with a 4 × 1 electrode setting has been confirmed (Hartmann et al., 2020; Kuo et al., 2013; Nikolin et al., 2015).

**Fig. 1:**
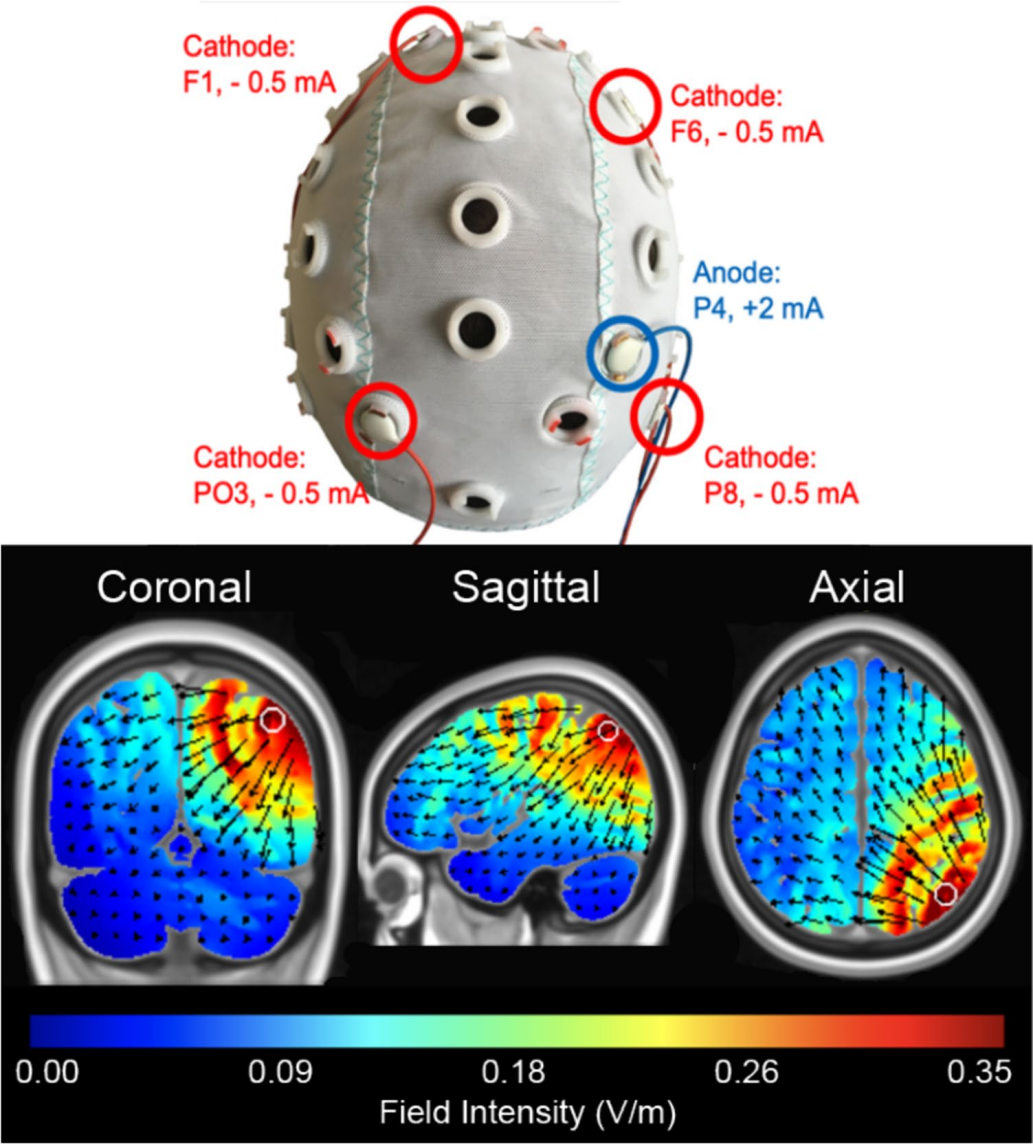
Electrode placement and simulated brain activity of HD-tDCS. The top panel shows the electrode placement for right parietal anodal HD-tDCS. The lower panel shows the result of the computer simulation for this setting for the coronal (left, right), sagittal (front, back), and axial (left, right) slice

Electrode placement was the same as in Hartmann et al. (2020). In order to stimulate the left and right IPS, the anodal electrode was placed over P3 and P4 of the international EEG 10–20-system (Klein et al., 2009, 2013). The optimal electrodes montage was confirmed by means of software (“HD-Explore”, “HD-Target”) that simulate the current flow into the brain depending on the given parameters (Soterix Medical, NY, USA). In a first step, electrode positions were determined by the software for selective maximal stimulation of BA 7, and then the proposed positions were manually modified in a way that led to the highest selective stimulation of the IPS (MNI coordinates 40, −64, 48 according to (Preuschhof et al., 2010)). These were the positions F5, F2, P7 and PO4 for left parietal stimulation, and F6, F1, P8 and PO3 for right parietal stimulation (see Fig. 1). This stimulation configuration resulted in an electric field over the target region (IPS) of 0.32 V/m, with lower electric field sizes in the surrounding areas (see Fig. 1). Previous studies reported tDCS effects for field sizes typically in the range of 0.3–0.4 V/m (Bikson et al., 2016), and some authors implied 0.2 V/m as threshold for neuronal interferences (Zito et al., 2015). We specifically assessed the electric field sizes in several other areas outside IPS, confirming that values were below 0.2 V/m (e.g., V1: < 0.12 V/m, visual association areas BA18, BA19: < 0.17 V/m, medial parietal BA 31: < 0.13).

The procedure of stimulation was the same as in Hartmann et al. (2020). At the onset of the stimulation, current was gradually increased over a period of 30 s until reaching the stimulation threshold of 2 mA (ramp-up phase). Following this, a constant direct current of 2 mA was applied for 25 min. Subsequently, the current was decreased to 0 mA over a 30-s period (ramp-down phase). In the sham condition, the same ramp-up procedure was applied, gradually increasing the current over 30 s until reaching the stimulation threshold. Once the threshold was reached, a full stimulation of 2 mA was delivered for 30 s, after which the current was ramped-down to 0 mA over another 30-s interval. The current remained off for the rest of the session. This procedure aimed to ensure that participants in both the real and sham stimulation condition experienced the initial tickling sensation of the current, rendering the two conditions indistinguishable (as validated by questionnaires, see the result section). For the sham condition, the left parietal stimulation setting was used for half of participants, and the right parietal stimulation setting for the other half. Impedance values were examined during the stimulation and were all below 10 kΩ for the duration of the entire session (typically around 4–7 kΩ).

**Fig. 2:**
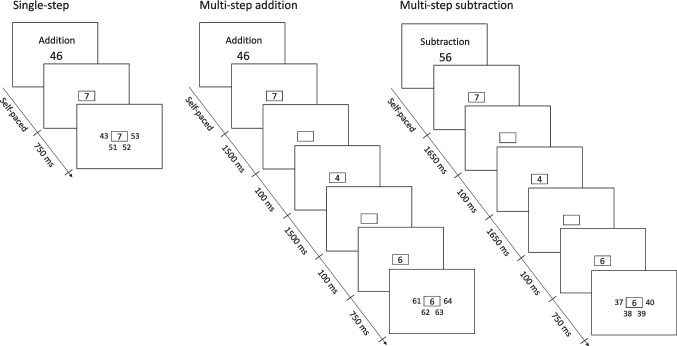
Example of trial sequences for a single-step addition (left), multi-step addition (middle) and multi-step subtraction problems (right)

Because tDCS is not effective at the beginning of stimulation (Nitsche & Paulus, 2000; Nitsche et al., 2008), participants started with the arithmetic task 5 min after stimulation onset. During the first 5 min of stimulation, participants performed 32 practice trials in which they trained the finger response mapping. Such a trial was identical to the screen of the arithmetic task where the four solution numbers were presented around the central box (see Fig. 2, last screen), except that the target number was an “X”, and the three lure cues were “O”. Participants then also performed 16 arithmetic practice trials before starting with the experimental trials. All participants completed these two practice blocks within 5 min after the onset of stimulation in all sessions. Participants completed the arithmetic task in about 15 min, and then started with the 2-back control task (see below), which took about 4 min. Thus, both tasks were completed during stimulation.

### Measures

#### **Arithmetic Task**

As outlined in the introduction, a key element of this study was the manipulation of both the operation type (addition, subtraction) and problem difficulty. Single-step problems, such as 23 + 4, involve only one computational step, similar to tasks used in prior studies (Artemenko et al., 2015; Hauser et al., 2013; Klein et al., 2013; Mosbacher et al., 2020). In contrast, three-step problems (23 + 4 + 7 + 6) require more complex and prolonged computational efforts. These problems were adapted from a previous study unrelated to NIBS, which confirmed that three-step problems rely more on active computation and less on memory retrieval compared to single-step problems (Hartmann, 2022). In each session, participants solved 96 arithmetic problems, comprising 24 single-step addition, 24 single-step subtraction, 24 multi-step addition, and 24 multi-step subtraction problems, presented in random order (see Table 2 in the Appendix for a list of problems). Participants were instructed to prioritize both speed and accuracy in selecting the correct solution. Each trial started with a screen displaying information regarding the operation (addition or subtraction) and the initial number (i.e., the “starting number”). The starting number ranged between 22 and 78 for addition problems, and between 32 and 88 for subtraction problems. Participants initiated the computation process by pressing the “space” key when prepared. The operands (one for single-step problems, three for multi-step problems) were presented sequentially in a square in the center of the screen (see Fig. 2). Operands were either 4, 5, 6, or 7. Operands of different magnitudes were used within the same trial, and the final result of a single-step problem, or the intermediate result of a multi-step problem, never yielded a multiple of ten. Across all trials within each condition (single-step addition, single-step subtraction, multi-step addition, and multi-step subtraction), the average operand size remained constant. Trial sequences are illustrated in Fig. 2. Since we expected greater difficulty of subtraction problems compared to addition problems, operands in multi-step subtraction problems were presented for a slightly longer duration (+ 150 ms) than in multi-step addition problems. This additional time was aimed at preventing the speed of the sequentially presented operands for multi-step subtraction problem from being too high.

After the presentation of the last operand, four potential solution numbers were displayed in a semicircular arrangement around the center of the screen, with ascending magnitudes from left to right (see Fig. 2). To correspond with the spatial layout of the solution numbers on the screen, participants positioned their left-hand index and middle fingers on the keys “v” and “f”, and their right-hand index and middle fingers on the keys “n” and “j”. The ascending alignment of the potential solution numbers in combination with the spatially congruent finger placement allowed for a straightforward selection of responses. The incorrect solutions deviated from the correct solution by ± 1, ± 2, ± 10, ± 11, or ± 12. The deviations of the three incorrect solution numbers were modified from trial to trial, ensuring that participants could not employ shortcuts or strategic decision-making (e.g., selecting the number that matches only in parity status, decade, or the units digit of the correct solution). To encourage participants to select the potential solution number only after they have finished the computation, a time delay of 750 ms was induced between the last operand and the four potential solution numbers. (Presenting the solution numbers simultaneously with the last operand might prevent participants from finishing their ongoing computation and intuitively select the best fitting solution.). The correct solution was presented equally often at each of the four possible positions. An error feedback (“wrong”) was displayed following an incorrect response for a duration of 2500 ms. After correct response or the display of the error message, the subsequent trial commenced after a blank screen of 1000 ms.

Three distinct sets of problems were created for the three sessions (referred to as Set A, B, and C). Each set contained a minimum of 85% unique problems, and no problem was presented in all three sets, ensuring minimal repetition and a diverse range of problems throughout the three sessions. The mean starting number, mean operand size, and mean result size were kept constant across the three problem sets. This ensured that participants encountered similar levels of complexity and difficulty regardless of the set they were assigned. An example of a stimulus set is shown in Table 2 in the Appendix. The assignment of problem sets, stimulation condition and session was counterbalanced across participants. The experiment was run on an ordinary laptop using PsychoPy2 (Peirce et al., 2019).

#### **Control task**

Considering the involvement of the PPC in domain-general functions like working memory and attention (Lazzaro et al., 2022), it is important to assess the extent to which any performance improvements due to IPS stimulation are specific to arithmetic processing (Looi & Cohen Kadosh, 2016). Therefore, we incorporated a n-back working memory task (Braver et al., 1997) as control task similar to Mosbacher et al. (2020). The n-back task entails processing number symbols, maintaining and updating a sequence of numbers in working memory, necessitating attentional resources, and requiring a motor response. Thus, this task shares several similarities with the arithmetic task, excluding the critical computational aspect. In the n-back task, participants were instructed to press the “j” key with the index finger of their right hand as quickly as possible if the number on the screen was the same as two numbers before (*n* = 2-back; henceforth referred to as the 2-back task). A total of 100 numbers from 1–9 were presented in pseudo-random order, so that a total of 20 hits were included in the task. As the operands in the arithmetic task, numbers in the n-back task were presented in the center of the screen inside of a box of 3.5 × 2°of visual angle. Each number was presented for 1000 ms, with an ISI of 550 ms. If participants missed a target or incorrectly pressed the button, the central box turned into red for 500 ms.

#### **Discomfort, pain, and condition guessing measurement**

As in Hartmann et al. (2020), participants were asked at the end of each session to indicate on a paper–pencil questionnaire how comfortable/uncomfortable the stimulation appeared to them (ranging from −3 = very uncomfortable to 3 = very comfortable), and how much pain they perceived during the stimulation (ranging from 0 = no pain to 10 = very strong pain). Moreover, after the last session, participants were informed that real brain stimulation was applied only in two out of the three sessions, and that a control stimulation (sham) was applied in one of the sessions. They were asked to guess which of the three sessions was the sham condition, and to indicate their confidence in this guess (ranging from 1 = very unsure to 5 = very sure).

### Data analysis

#### **Response times (RTs)**

RT analysis was based on correct trials (85.0%). Data trimming was performed in a two-step procedure as described by Mann et al. (2011). In a first step, responses shorter than 200 ms and longer than 5000 ms for single-step, or 8000 ms for multi-step problems were excluded. In a second step, all RTs deviating from the individual’s condition mean (step size x stimulation x operation) by more than 2.5 *SD* were excluded. This procedure was repeated iteratively until no more outliers were detected. RTs were log-transformed for analysis to normalize their distribution, and untransformed means are reported. RTs reflect the time between the onset of the solution and participant’s response.

RTs were analyzed by means of a repeated measure linear mixed-effects model using the lme4-package in R (Bates et al., 2015). The model included the fixed effect predictors stimulation (left, right, sham), operation (addition, subtraction), step size (single, multi), and all interactions of these three predictors. We also added the fixed effect predictor session (1, 2, 3) to account for training effects across sessions. Session was defined as ordered factor (Session 1 < Session 2 < Session 3), thus a significant effect of session indicates that participants improve over sessions. Given the repeated measure design, a random intercept for participant was added, as well as a random slope of stimulation per participant. Lastly, a random intercept effect was added for each problem in order to account for variance in RTs that were caused by the specific problems within each category of operation and step size (see Artemenko et al., 2015 for a similar approach). Statistical report was based on Type III Analysis of Variance (ANOVA) using Satterthwaite approximation for the calculation of degrees-of-freedom (Luke, 2017). Since a standard procedure for calculating effect sizes for linear mixed-effects models is not yet fully established, we reported the standardized estimates for the model coefficients as a measure of effect size (see also Artemenko et al., 2015). Standardized estimates express the relationship between predictors and the outcome variable in terms of standard deviations, making them easier to interpret, particularly when comparing the relative importance of predictors. These standardized estimates are meaningful for continuous predictors and for two-level categorical predictors (in our case operation, step size, and session), where they indicate the effect size of moving from one level to the other. For categorical predictors with more than two levels, such as our three-level predictor HD-tDCS stimulation, standardized estimates cannot be computed for the overall effect but can be derived for pairwise comparisons between two specific levels (left vs. sham, left vs. right, sham vs. right). To obtain standardized estimates for these pairwise comparisons, we refitted the models using different reference categories. The lme-4 analysis syntax and the normal quantile–quantile plot of the model confirming normality of residuals can be found in the Supplementary Information.

#### **Accuracy**

Given that tDCS effects on mental arithmetic are typically observed at the level of RTs—likely by increasing the speed with which numerical information is processed—and not at the level of accuracy (Artemenko et al., 2015; Hauser et al., 2013; Kasahara et al., 2013; Klein et al., 2013; Mosbacher et al., 2020), we did not expect HD-tDCS to impact the accuracy of arithmetic problem solving. However, for the sake of completeness, we also analyzed the error rate by means of a logistic regression mixed-effects model with 1 = correct and 0 = error, using the same model specifications as described for RTs. As a measure of effect size for the logistic regression, we provided the odds ratio. The odds ratio compares the odds of an outcome (in this case a correct response) occurring in one condition to the odds of it occurring in another. An odds ratio greater than 1 indicates that the event is more likely to occur in the first condition, while an OR less than 1 suggests it is less likely. An OR of 1 implies no difference in odds between conditions.

#### **Control task**

For the 2-back task, log RTs of correctly detected targets and accuracy were analyzed. For the analysis of log RTs, outliers were removed using the same procedure as described for the arithmetic task. Since there were no trial level predictors, log RTs were averaged for each participant and stimulation condition and analyzed by means of a linear mixed-effects model with the fixed effects of stimulation (left, right, sham) and the ordered factor session (1, 2, 3), with a random intercept for participant.

The mean accuracy over all 100 trials was aggregated for each participant and stimulation condition, and analyzed with the same linear mixed-effects model as described for RTs. Statistical report was based on Type III Analysis of Variance (ANOVA) using Satterthwaite approximation for the calculation of degrees-of-freedom. Again, we reported standardized estimates as measure of effect size. The lme-4 analysis syntax and the normal quantile–quantile plot of the model confirming normality of residuals can be found in the Supplementary Information. Data from one session was missing due to technical problems.

## Results

### Arithmetic task

A total of 7,200 trials were generated from the arithmetic task, with 6,117 correct responses (85.05%) and 1,083 incorrect responses (14.95%). From the correct responses, 305 trials were removed during the first step of data trimming, and an additional 45 trials were removed in the second step (see Data analysis). This resulted in 5,767 trials being used for the analysis of RTs.

#### **RTs**

**Fig. 3:**
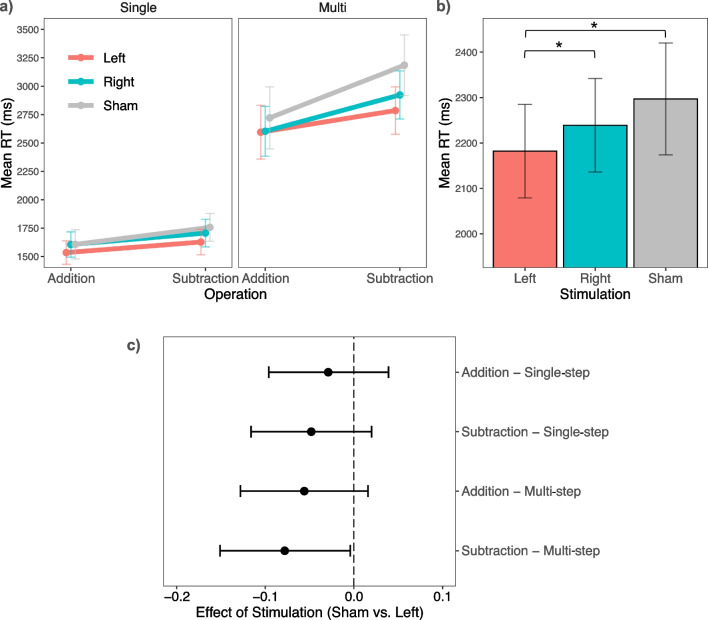
Results of the arithmetic task. Mean RTs for each type of arithmetic problem (**a**) and averaged for each stimulation condition (**b**). Asterisks indicate significant pairwise comparison (*p* < 0.05), and error bars show ± 1 *SEM*. (**c**) shows the effect of stimulation (log difference between sham and left IPS stimulation) for each type of arithmetic problem. Error bars represent 95 CI

Mean RTs for each condition (addition – single step, subtraction – single step, addition – multi step, subtraction – multi step) are illustrated in Fig. 3a, and the ANOVA results of the linear mixed-effects model analysis are summarized in Table 1. As expected, there was a main effect of operation, *F*(1,276.8) = 18.59, *p* < 0.001, std. estimate = −0.10, *SE* = 0.02, and a main effect of step size,* F*(1,277.1) = 196.47, *p* < 0.001, std. estimate = 0.32, *SE* = 0.02. Participants were faster when solving addition (*M* = 2111, *SEM* = 88) than subtraction (*M* = 2331, *SEM* = 91) problems, and they were faster when solving single (*M* = 1640, *SEM* = 47) than multi step (*M* = 2802, *SEM* = 97) problems. Most importantly, there was a main effect of stimulation, *F*(2,23.9) = 3.64, *p* = 0.042. Pairwise comparison showed that participants were significantly faster in arithmetic problem solving during left (*M* = 2136, *SEM* = 103) when compared to sham stimulation (*M* = 2318, *SEM* = 123, *p*_left vs. sham_ = 0.017, std. estimate = −0.08, *SE* = 0.03),^3^ and also faster when compared to right stimulation (*M* = 2210, *SEM* = 103, *p*_left vs. right_ = 0.042, std. estimate = −0.05, *SE* = 0.03), as illustrated in Fig. 3b. There was no difference between sham and right stimulation (*p* = 0.391, std. estimate = −0.02, *SE* = 0.03). The additional main effect of session, *F*(2, 43.7) = 73.54, *p* < 0.001, std. estimate = −0.21, *SE* = 0.02, indicated that participants became faster over the three sessions (Session 1: *M* = 2481, *SEM* = 119; Session 2: *M* = 2180, *SEM* = 110; Session 3: *M* = 2002, *SEM* = 95). None of the interactions was significant. We also plotted the effect size of stimulation (difference in log RTs between left stimulation and sham) for each condition in Fig. 3c. This additional figure shows that the single-step addition contributed the least, while the multi-step subtraction problems contributed the most to the effect of stimulation.

**Table 1:** ANOVA table for the linear mixed-effects model (RTs) and the linear mixed-effects logistic regression model (accuracy)

	RT		Accuracy	
Effect	*F*	*p*	*F*	*p*
Stimulation	3.64	0.042	0.27	0.767
Operation (Op)	18.59	< 0.001	0.57	0.450
Step size (Ss)	196.47	< 0.001	108.92	< 0.001
Session	73.54	< 0.001	11.82	< 0.001
Stimulation x Op	0.39	0.679	0.50	0.604
Stimulation x Ss	0.59	0.554	1.62	0.198
Op x Ss	3.70	0.055	< 0.00	0.967
Stimulation x Ss	0.64	0.53	1.62	0.198
Stimulation x Op x Ss	0.01	0.996	0.67	0.510

Type III ANOVA table with Satterthwaite’s method

Additional analysis of RTs for single-step problems accounting for the additional variable carry can be found in the Supplementary Information.

#### **Accuracy**

Results are summarized in Table 1. There were significant main effects of step size,* F*(1,279.94) = 108.92, *p* < 0.001, odds ratio = 0.56, and session,* F*(2,40.74) = 11.82, *p* < 0.001, odds ratio = 1.38. Participants solved more single-step (*M* = 91.1%, *SEM* = 0.7) than multi-step (*M* = 78.8%, *SEM* = 1.1) problems correctly, and they improved over sessions (Session 1: *M* = 82.3%, *SEM* = 1.4; Session 2: *M* = 85.5%, *SEM* = 1.2; Session 3: *M* = 87.1%, *SEM* = 1.2). As expected, there was no main effect or interaction effects of stimulation (all *p*’s > 0.202). There was also no main effect of operation, *F*(1, 281.22) = 0.48, *p* = 0.490, odds ratio = 1.05 (addition: *M* = 85.4%, *SEM* = 1.0; subtraction: *M* = 84.6%, *SEM* = 1.1).

### Control task

One trial with an RT of less than 200 ms was excluded in the first step, and an additional 6 trials were excluded for deviating more than 2.5 SD from the individual mean per session in the second step of data trimming.

#### **RTs**

**Fig. 4:**
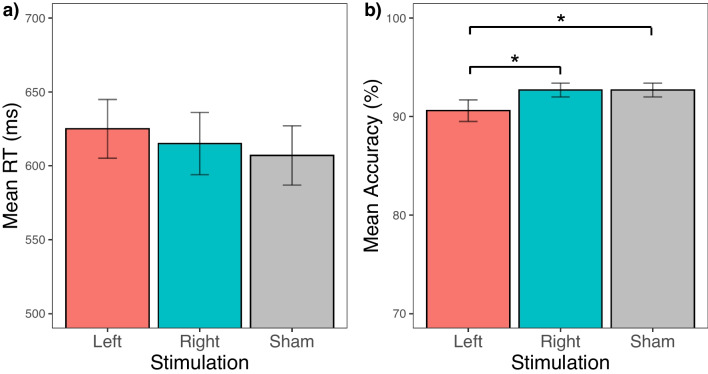
Results of the 2-back control task. Mean RTs (**a**) and accuracy (**b**) for each stimulation condition. Asterisks indicate significant pairwise comparison (*p* < 0.05), and error bars show ± 1 *SEM*

The linear mixed-effects analysis revealed no effect of stimulation, *F*(2, 45.27) = 0.35, *p* = 0.709 (left: *M* = 625, *SEM* = 20; right: *M* = 615, *SEM* = 21; sham: *M* = 607, *SEM* = 20) (see Fig. 4), and participants did not become faster over sessions, *F*(2, 45.27) = 1.44, *p* = 0.247, std. estimate = −0.18, *SE* = 0.12 (Session 1: *M* = 633, *SEM* = 19; Session 2: *M* = 609, *SEM* = 21; Session 3: *M* = 605, *SEM* = 20).

#### **Accuracy**

The linear mixed-effects analysis revealed a main effect of stimulation, *F*(2, 44.48) = 4.24, *p* = 0.021, and also a main effect of session, *F*(2, 44.48) = 5.36, *p* = 0.008, std. estimate = 0.46, *SE* = 0.14. Pairwise comparison revealed that participants were less accurate during left stimulation (*M* = 90.6%, *SEM* = 1.1) when compared to sham stimulation (*M* = 92.7%, *SEM* = 0.7, *p*_left vs. sham_ = 0.017, std. estimate = −0.50, *SE* = 0.20) and also less accurate when compared to right stimulation (*M* = 92.7%, *SEM* = 0.7, *p*_left vs. right_ = 0.014, std. estimate = −0.53, *SE* = 0.21), as shown in Fig. 4. There was no difference between sham and right stimulation (*p* = 0.904, std. estimate = 0.03, *SE* = 0.21). Participants improved accuracy over the three sessions (Session 1: *M* = 90.6%, *SEM* = 0.9; Session 2: *M* = 92.2%, *SEM* = 0.8; Session 3: *M* = 93.4%, *SEM* = 0.08).

### Discomfort, pain, and condition guessing measurement

#### **Discomfort**

A repeated measures ANOVA with the variable stimulation (left, right, sham) revealed no difference in discomfort (1 = very comfortable, 7 = very uncomfortable) across conditions, *F*(2,48) = 1.42, *p* = 0.251, η^2^_p_ = 0.06 (*M*_Left_ = 4.68, *SEM* = 0.17; *M*_Right_ = 4.60, *SEM* = 0.18; *M*_Sham_ = 4.44, *SEM* = 0.20).

#### **Pain**

The same analysis on the mean pain score revealed a significant difference in perceived pain (0 = no pain, 10 = unable to move) across stimulation conditions, *F*(2,48) = 5.30, *p* = 0.008, η^2^_p_ = 0.18. Pain ratings during sham were significantly lower when compared to left (*p* = 0.025) or right (*p* = 0.008) stimulation, but mean pain ratings were generally low and varied between 1 (minimal) and 2 (mild) (*M*_Left_ = 1.56, *SEM* = 0.25; *M*_Right_ = 1.68, *SEM* = 0.25; *M*_Sham_ = 1.04, *SEM* = 0.19).

#### **Condition guessing**

When asked at the end of the third session to guess post-hoc which of the three sessions was the sham condition, 13 out of 25 participants correctly guessed the sham session. We performed a proportion test using the prop.test-function from base R (v4.4.1: R Core Team, 2024) to evaluate whether the observed proportion of success (13/25 = 0.52) is significantly different from the expected proportion (correctly guessing chance rate of 1/3). The test did not reveal a significant difference between observed and expected proportion, χ^2^ (*df* = 1, adjusted for small sample sizes) = 3.13, *p* = 0.078. Moreover, there was no difference in confidence between participants who correctly indicated the sham session and those who did not (*M*_correct_ = 2.92, *SEM* = 0.45; *M*_incorrect_ = 2.42, *SEM* = 0.38), as revealed by independent sample *t*-test (*p* = 0.334).

## Discussion

NIBS techniques such as tDCS hold significant promise as a tool for both investigating the functional involvement of specific brain areas in mental arithmetic and also for increasing arithmetic performance. Previous studies have yielded inconsistent findings, necessitating further investigation. Therefore, the objective of this study was to shed light on the role of the left and right IPS in mental arithmetic using HD-tDCS, which, to the best of our knowledge, had not been employed for this purpose previously.

Across both single- and multi-step addition and subtraction problems, we observed that HD-tDCS applied over the left IPS resulted in increased speed of arithmetic performance. Notably, stimulation of the right IPS did not yield any significant benefits. Consequently, our results provide further evidence that the IPS serves as a fundamental hub for number representation and arithmetic (e.g., Arsalidou & Taylor, 2011; Dehaene, 2011; Knops, 2017; Piazza et al., 2007), and more specifically that the left IPS is functionally involved in arithmetic problem solving across a broad range of problem types. The observed selective effect of left IPS stimulation aligns with previous NIBS studies that have reported left hemispheric parietal specialization in number processing and arithmetic (e.g., Andres et al., 2005; Grabner et al., 2015; Hauser et al., 2013; Kasahara et al., 2013; Pesenti et al., 2000; Sasanguie et al., 2013).

Impairments in number processing, such as dyscalculia, have been associated with both functional and structural abnormalities in the fronto-parietal network (De Smedt et al., 2019). Our finding that the IPS is responsive to HD-tDCS highlights the potential of electrical brain stimulation as a tool to enhance cognitive performance in individuals with such impairments (Cohen Kadosh et al., 2010; De Smedt et al., 2019; Lazzaro et al., 2022). However, it is important to acknowledge that our sample included only participants without self-reported number processing impairments, limiting the generalizability of the results to individuals with such impairments. Additionally, we did not include any measures to rule out dyscalculia or assess math-related abilities (e.g., math skills, IQ) in the present study. Therefore, it remains to be examined whether HD-tDCS-induced improvements extend to individuals with dyscalculia, or to what extent general math skills in non-dyscalculic individuals may moderate the effects of electrical brain stimulation.

Our initial hypothesis suggested that the dependency on the left IPS would vary according to the complexity of the arithmetic problem. However, our results did not support this hypothesis, indicating that the effect of left IPS stimulation may not depend as strongly on specific characteristics of arithmetic problems as we had anticipated. Notably, the present study likely lacked sufficient statistical power to detect small higher-order interaction effects, so the potential moderating role of task difficulty on stimulation-induced gains in arithmetic processing still warrants further investigation. Examination of effect sizes for the different types of problems (Fig. 3c) revealed that single-step addition problems contributed the least to the facilitatory effect of left IPS stimulation, while the most challenging multi-step subtraction problems contributed the most. This descriptive observation suggests that more difficult arithmetic problems involving an extended computation phase may potentiate the effects of left IPS stimulation. It is possible that complex tasks require multiple access to number representation, thereby benefiting more from enhanced access to this representation. This finding may help explain why previous studies utilizing single-step problems have not consistently observed an effect of IPS stimulation on arithmetic performance (Hauser et al., 2016; Mosbacher et al., 2020, 2021), or why the facilitatory effect of left IPS stimulation has sometimes been restricted to more complex problems (Rütsche et al., 2015; see also Salillas et al., 2012). Consistent with this idea, a previous tRNS-assisted cognitive training study highlighted that task difficulty might be an important modulator of stimulation-induced performance gains (Popescu et al., 2016).

To assess the specificity of the effects of HD-tDCS on arithmetic processing, we also evaluated performance on a control 2-back task. Unexpectedly, left HD-tDCS led to poorer accuracy compared to right stimulation or sham. Since the effect of stimulation was opposite to that observed in the arithmetic task, this suggests that the improvement in arithmetic performance is not due to a task-unspecific enhancing effect, such as improved attention or working memory (as this would have resulted in improvements in both tasks). The detrimental effect of left IPS stimulation on the control task may indicate the induction of unwanted inhibitory effects. In the left anodal stimulation condition, one of the cathodal electrodes was positioned at PO3, in the parieto-occipital area of the right hemisphere (see Fig. 1). Previous research has shown that the right parietal lobe plays a key role in supporting working memory (Berryhill et al., 2010; Jones & Berryhill, 2012; Koenigs et al., 2009). Thus, inhibition from the cathodal electrode could explain these findings. We introduced HD-tDCS as a method to stimulate target brain areas more precisely while minimizing the unwanted inhibitory effects of cathodal stimulation, which are more common in traditional tDCS setups. However, given the inhibitory effects observed in the control task and considering the field intensity simulation (Fig. 1), we cannot rule out the possibility that our HD-tDCS setup still caused spill-over effects or induced inhibition in distant areas. Future studies might consider using a weaker current to reduce such potential confounds and fully exploit the advantages offered by HD-tDCS. Additionally, counterbalancing the order of the experimental and control tasks could help make the effects of stimulation more comparable.

In the arithmetic task, we included a time delay between the last operand and the solution. A time delay was induced to encourage participants to continue the mental arithmetic process (cf. stimuli and task procedure). The duration of the delay was determined based on a pretest and was set to be rather short (750 ms), but we cannot rule out that in some trials, participants may have already solved the problem before the solution numbers appeared, particularly when they quickly retrieved the solution from memory. This might reduce the likelihood to find a subtle facilitatory effect of IPS stimulation for quickly solved problems. Since there was a selective enhancing effect of left but not right IPS stimulation, and since all type of problems contributed to this effect to some extent (Fig. 3c), we do not think that this methodological issue had a critical effect on our results. However, it limits the conclusions about a possible moderating effect of problem difficulty, which should be further addressed in future research.

Furthermore, our experimental approach did not allow us to disentangle the specific effects of IPS stimulation on number representation and numerical computation. It is plausible that the observed effects were driven by an enhanced access to number meaning, an augmentation of mental manipulation processes, or a combination of both. To gain a deeper understanding of these underlying mechanisms, future research should consider integrating IPS stimulation with neuroimaging techniques such as fMRI or electroencephalography (EEG). A combination of these approaches would enable the exploration of neural processes and provide valuable insights into the precise mechanisms involved in IPS stimulation effects (Hauser et al., 2016; Mosbacher et al., 2020; Rütsche et al., 2015). For example, Rütsche et al. (2015) discovered that the effects of IPS stimulation were associated with modulations of oscillatory EEG activity in different frequency bands. Specifically, they found distinct patterns of modulation in the alpha band related to procedural arithmetic processes, while modulations in the theta band were linked to memory-based arithmetic processes. Furthermore, they observed that larger arithmetic problems were associated with increased lower-alpha desynchronization, whereas smaller problems resulted in decreased theta synchronization. These findings indicate a differential engagement of the left IPS in arithmetic problem-solving of varying complexity. This underscores the considerable variability in brain stimulation effects based on the specific neuro-cognitive processes involved. Integrating NIBS with neuroimaging methods might also provide valuable insights not only into the different effects of individual brain regions, such as the IPS, but also into the broader cortical network (Pisoni et al., 2018).

The assessment of discomfort and pain in our participants further confirms the tolerability of the stimulation settings used in this study. Notably, around half of the participants correctly guessed the sham condition, albeit with low confidence. This underscores the importance of participants being unaware of the specific expectations related to the stimulation effects.

In this study, the task was fully standardized, and no interaction between the experimenter and participant was necessary, minimizing the risk of experimenter-induced bias. However, future studies could implement double-blind designs to eliminate any potential bias completely.

Despite the aforementioned limitations, we believe that this study makes a significant contribution to the growing body of evidence supporting the functional involvement of the left IPS in mental arithmetic, and it provides further evidence for the potential of electrical brain stimulation to enhance arithmetic performance. Previous studies have demonstrated that electrical brain stimulation applied to the IPS, including tRNS, can improve mathematical learning (Lazzaro et al., 2023; Mosbacher et al., 2021; Pasqualotto, 2016; Popescu et al., 2016; Snowball et al., 2013). With the current study establishing performance improvements through left IPS stimulation using HD-tDCS, it opens up exciting avenues for exploring the use of HD-tRNS as a tool to enhance arithmetic learning, potentially leading to more effective and targeted interventions.

## Electronic supplementary material

Below is the link to the electronic supplementary material.

Supplementary file1 (DOCX 535 KB)

## Data Availability

Data and analysis code are available at: https://osf.io/f2jgn (analysis code is in the Supplementary Information file). We confirm that we have reported all measures, conditions, data exclusions and information regarding the sample size used in this study. Analysis code can be found in the Supplementary Information file.

## Appendix

**Table 2:** One-step and multi step arithmetic problems

Addition		Subtraction	
Single-step	Multi-step	Single-step	Multi-step
22 + 4	23 + 4 + 7 + 6	32—4	33—4—7—6
23 + 4	24 + 5 + 7 + 6	33—6	34—5—7—6
27 + 5	26 + 5 + 6 + 7	37—5	36—5—6—7
28 + 5	27 + 4 + 6 + 7	38—5	37—4—6—7
32 + 6	33 + 5 + 7 + 4	42—6	43—5—7—4
33 + 6	34 + 5 + 6 + 4	43—7	44—5—6—4
37 + 6	36 + 6 + 4 + 5	47—6	46—4—6—5
38 + 6	37 + 4 + 7 + 5	48—6	47—4—7—5
42 + 6	42 + 7 + 5 + 6	52—6	52—6—7—5
44 + 7	44 + 7 + 6 + 4	54—7	54—7—6—4
46 + 7	46 + 7 + 4 + 6	56—7	56—7—4—6
48 + 4	48 + 7 + 6 + 5	58—4	58—7—6—5
52 + 7	52 + 6 + 5 + 4	62—6	62—6—5—4
54 + 7	54 + 7 + 4 + 5	64—7	64—7—4—5
56 + 7	56 + 7 + 5 + 4	66—7	66—7—5—4
58 + 7	58 + 4 + 5 + 6	68—7	68—4—5—6
63 + 5	62 + 6 + 4 + 7	73—5	72—6—4—7
64 + 5	63 + 6 + 7 + 5	74—5	73—7—5—6
66 + 6	67 + 6 + 5 + 7	76—4	77—6—5—7
67 + 4	68 + 6 + 7 + 4	77—4	78—6—7—4
73 + 4	72 + 4 + 5 + 7	83—4	82—4—5—7
74 + 5	73 + 4 + 6 + 5	84—5	83—6—4—5
76 + 5	77 + 5 + 4 + 6	86—5	87—5—4—6
77 + 4	78 + 5 + 4 + 7	87—4	88—5—4—7

## Footnotes

1 “Symbolic mental arithmetic” refers to arithmetic operations that involve the use of Arabic numerals (as is common in Western cultures) as symbols for numbers. In contrast, “non-symbolic arithmetic” involves the use of non-numerical representations, such as a set of visual items like dots, to represent quantities.

2 Note that this a priori sample size estimation using G*Power is only an approximation, as we employed a more complex (2 × 2 × 3) linear mixed-effects model, for which there is no standard procedure to compute exact a priori sample sizes. Linear mixed-effects models typically offer greater statistical power compared to conventional ANOVAs or t-tests, which are based on aggregated mean values across trials. This increased power arises because mixed-effects models account for both fixed and random effects at the subject and trial level, thus modeling variability from multiple sources more efficiently (Baayen et al., 2008).

3 At the individual level, 15 out of 25 participants (60%) showed faster responses during left compared to sham stimulation. A figure depicting the effect of stimulation (left – sham) for each participant can be found in the Supplementary Information (Figure SI 2).
