# Supplementary material for: Anodal High-definition Transcranial Direct Current Stimulation Over the Left (but not Right) Parietal Cortex Facilitates Mental Arithmetic
Source: J Cogn Enhanc. 2024 Dec 4;9(1):51–66. doi: 10.1007/s41465-024-00314-0 (PMC11914294; doi:10.1007/s41465-024-00314-0)
Supplement: Supplementary file 1 — Supplementary file1 (DOCX 535 KB) [file 41465_2024_314_MOESM1_ESM.docx]

Supplementary Information

**Anodal high-definition transcranial direct current stimulation over the left (but not right) parietal cortex facilitates mental arithmetic**

Matthias Hartmann^1,2^, Magali Dumureau^1^

Journal of Cognitive Enhancement

^1^Faculty of Psychology, Swiss Distance University Institute, Switzerland

^2^Department of Psychology, University of Bern, Switzerland

Data analysis syntax (lme4) and normal quantile-quantile plots

Data are available at: <https://osf.io/f2jgn/>

**Arithmetic task**

**Analysis of response times**

| Figure SI 1  Normal quantile-quantile plot (arithmetic task, RTs) |
| --- |
|  |

Model:

Arithmetic_RT.m = Model syntax: lmer(logRT ~ Operation*Stimulation*StepSize + Session + (1 +Stimulation | participant) + (1 |TrialID), data = Arithmetic_RT, control = lmerControl(optimizer ="optimx",optCtrl = list(method = "nlminb")))

Show results of the model:

anova(Arithmetic_RT.m)

*Note: This analysis is based on correct responses without outliers (see data analysis section for more information)*

| Figure SI 2  Individual effects of stimulation (Sham – Left) in ascending order |
| --- |
|  |

*Note.* Each bar represent one participant.

**Additional analysis of RTs: accounting for carry effects in single-step problems**

For the analysis of single-step problems reported in the article, problems with carry (e.g., 46 + 7 or 64 – 7) and without carry (e.g., 23 + 4 or 87 – 4) were analyzed together. Problems with carry are more complex and rely more strongly on procedural processes than problems without carry. To additionally assess whether carry moderated the effect of stimulation for the single-step problems, we repeated the analysis described above for single-step problems adding carry and all higher order interactions between carry, operation and stimulation as additional fixed effects. The separate analysis of single-step problems confirmed an effect of stimulation, although slightly below significance threshold, *F*(2,3039.67) = 2.89, *p* = .056 (*M*_Left_ = 1544, *SEM* = 63, *M*_Right_ = 1623, *SEM* = 69, *M*_Sham_ = 1654, *SEM* = 73), an effect of carry, *F*(1,136.83) = 121.19, *p* < .001 (*M*_Carry_ = 1893, *SEM* = 56, *M*_NoCarry_ = 1321, *SEM* = 44), an effect of operation, *F*(1,136.82) = 4.93, *p* = .028 (*M*_Addition_ = 1554, *SEM* = 53, *M*_Subtraction_ = 1660, *SEM* = 58), and an effect of session, *F*(2,2931.16) = 46.94, *p* < .001 (*M*_Session1_ = 1755, *SEM* = 71, *M*_Session2_ = 1596, *SEM* = 71, *M*_Session3_ = 1470, *SEM* = 59). Most importantly, there was no interaction between carry and stimulation, *F*(2,3039.43) = 0.59, *p* = .552, and no three-way interaction between carry, stimulation and operation, *F*(2,3039.58) = 1.16, *p* = .313.

**Analysis of errors**

Model:

Arithmetic_errorrate.m = glmer(Correct ~ Operation*Stimulation*StepSize + Session + (1 + Stimulation | participant) + (1|TrialID), family = binomial(link='logit'), data = Arithmetic_errorrate, control = glmerControl(optimizer="bobyqa", optCtrl = list(maxfun=2e5)))

Show results of the model:

afex::mixed(Arithmetic_errorrate.m, data = Arithmetic_errorrate)

*Note: Normal quantile-quantile plot is useless for logistic regression models*

**Control task (2-back)**

**Analysis of response times**

| Figure S3  Normal quantile-quantile plot (control task, RTs) |
| --- |
|  |

Model:

NBack_RT.m = lmer(RTLog ~ Stimulation + Session + (1 | participant), data = NBack_RT)

Show results of the model:

anova(NBack_RT.m)

*Note: since there were no trial-level predictors, this analysis was performed on aggregated data. This analysis is based on correct responses without outliers (see data analysis section for more information).*

**Analysis of Accuracy**

| Figure S4  Normal quantile-quantile plot (control task, accuracy) |
| --- |
|  |

Model:

NBack_accuracy.m = lmer(Accuracy ~ Stimulation + Session + (1|participant), data = NBack_accuracy)

Show results of the model:

anova(NBack_accuracy.m)

*Note: since there were no trial-level predictors, this analysis was performed on aggregated data.*
